# Health and quality of life outcomes

**DOI:** 10.1186/s12955-014-0173-5

**Published:** 2014-12-12

**Authors:** Meenakshi Jolly, Winston Sequeira, Joel A Block

**Affiliations:** Department of Medicine/Rheumatology, Rush University Medical Center, 1611 W Harrison Street, suite 510, Chicago, IL 60612 USA

**Keywords:** ■■■

## Dear editors

We read with interest the recently published paper titled *Patient-Reported Outcome Measures for Systemic Lupus Erythematosus Clinical Trials: a Review of Content Validity and Psychometric Performance* [1]. We applaud the authors for undertaking this study to identify the relative strengths and weaknesses of patient reported outcome (PRO) tools being currently used in systemic lupus erythematosus (SLE).

We feel that some of the information provided on LupusPRO is inadvertently misleading, while some pertinent information on SF36 and LupusQoL may have been not discussed. The conclusions thus drawn may undermine the utility of LupusPRO and favorably bias the readers towards SF36 or LupusQoL.

Conceptual content validity: The authors note that the current literature is lacking on evidence on importance of concepts related to “treatment satisfaction”, “adherence” and “impact of flares” and are thus “future considerations”. LupusPRO [2] included all three items in the item pool derived from patient feedback. “Treatment satisfaction” and “Flares” are represented in the “Satisfaction with Care” and “Lupus Symptoms” domains. Adherence tied closely with medications and their side effects, is represented in “Lupus Medications” domain. Furthermore, we note several concepts that LupusPRO covers, but were not reported in Table two. These include “marks on skin”, and “sleep”. “Leisure activities” is reflected in LupusPRO item “kinds of tasks or activities I could perform”, while “weight gain”, “looking sick” are captured in LupusPRO body image items on “appearance changes”. LupusPRO thus reflects 14/27 concepts listed in the table. Additionally, LupusPRO identifies with other concepts, some identified by the authors (not listed in the Table), and include “fear that disease would change”, “control”, “caring for children”, “fulfilling their role in family”, ”progression of career”, “receiving support”, “ability to conceive or have children”, “flares”, “medication side effects” and ‘satisfaction with care”. Others have also identified trust in medical doctors, coping, family role functioning, chronicity of disease, drugs, family support, functions related to pregnancy, need for medications and side effects of medications to be important concepts for SLE [3,4]. Similar to LupusQoL, we did identify importance of relationships with family, friends, intimate partner and effects on sex. As the tool has undergone further validation studies, using clinimetric and psychometric approaches, these items were not be ranked highly and were dropped. Receiving support from partner/family or friends was considered more important and were retained. LupusPRO’s face validity has been tested since the initial publication, in two focus groups independently, during the development of the lupus impact tracker [5]. Other conceptual coverage errors noted in Table two pertain to conceptual coverage of “weakness”, “sleep”, “relationships”, “work disability” and “economic impact” by SF36. Hence, the generalization that SF-36, LupusQoL and SLEQOL demonstrated greatest level of conceptual coverage as compared to other tools seems erroneous.

Other psychometric properties: LupusPRO has excellent construct validity against EQ5D, SF-36, depression and body image tools in the original English version, as well as in various languages and regions [2,6-10]. It is responsive to change against self-reported change in health status [2], physician global assessment [2,11,12], BILAG [2], LFA defined flare [2], SLEDAI [11,12] and SELENA Flare Index [11,12]. SLEQOL, LupusPRO, LupusQoL [13,14] and SF-36 [15] have been noted to have high floor/ceiling effects (>15%). SF-36 has been reported to have high floor and ceiling effects in several diseases [16-18], and is used widely in research and clinical trials, including SLE. Infact, floor and ceiling effects of SF-36 were reported in the SLEQOL development study among SLE patients [15]. The authors should note that LupusQoL and LupusPRO have been cross culturally validated in several languages and regions, and continue to show fair psychometric properties. In addition, LupusPRO is the only tool thus far to demonstrate measurement equivalence of the tool across languages and regions [19]. The statement that only L-QoL and LupusQoL had sufficient evidence of validity for SLE population is incorrect. Perhaps the authors wished to indicate in the table is the floor effects, and not its construct validity.

Item formatting: Though the authors differ in opinion, we feel that wording of LupusQoL items 10, 11 and 15 of LupusQoL may be confusing to the patient about the concept/s being measured. For example, item 10, interference of sleep by pain from lupus is being sought. A patient may have difficulty ascribing causality of the pain to lupus, and then to their sleep. In addition, a lupus patient with sleep interference, but from lupus related fatigue, depression or anxiety may endorse the item.

A tool with low ceiling and floor effects is preferable; however, not every concept measured has a true hierarchy of difficulty or ability. Furthermore, the domain may be measuring the range of the concept that is appropriate for the selected population. We need further studies to explore and understand this issue. Greater collaboration would lead us to further patient reported outcomes research and clinical trials in SLE to improve patients overall outcomes and quality of life.

Sincerely

Meenakshi Jolly, MD

Winston Sequeira, MD

Joel A Block, MD
